
JOURNAL INFORMATION
==============================
NLM Title Abbreviation: Tex Med J (Austin)
NLM Journal ID: 21130590R

Texas Medical Journal

ISSN: 0892-8495
Publisher: Biomedical Journal Digitization Project

ARTICLE INFORMATION
==============================
PMCID: PMC9642699
PMID: 36957010
Article version: 1
Subjects: Abstracts and Selections

Control of Criminals and Defectives in New Jersey

Print publication date: 1912 Sep
Volume: 28
Issue: 3
First page: 125
Last page: 126
License: This work is free of known copyright restrictions. For more information, please see PMC Back Issue Digitization.
License URL: https://creativecommons.org/publicdomain/mark/1.0/

==============================

THE CHAS. H. PHILLIPS CHEMICAL CO.
NEW YORK and LONDON

**PHILLIPS' MILK OF MAGNESIA**

"THE PERFECT ANTACID"

SUPERIOR TO LIME WATER, BICARBONATE OF SODA, CHALK, ETC.
for correcting Hyperacid conditions—local or systemic.
Vehicle for Salicylates, Iodids, Balsams, etc.

PHILLIPS' REFINED CAMPHOR
PHILLIPS' WHEAT PHOSPHATES
PHILLIPS' DIGESTIBLE COCOA
PHILLIPS' PHOSPHO-MURIATE OF QUININE
PHILLIPS' SYRUP OF WHEAT PHOSPHATES
PHILLIPS' EMULSION OF COD LIVER OIL

the malady in question, together with the technique of administration, is now appearing in the commercial pages of the leading medical publications. Practitioners are advised to consult these current announcements.

---

Control of Criminals and Defectives in New Jersey.—Nearly half of the 600 inmates of the New Jersey reformatory are mentally defective, according to Dr. G. L. Orton, physician of the reformatory. He reports the results of two years' investigation in a recent number of the *Journal of the American Medical Association.* Following the system of Binet, the noted French psychologist, Dr. Orton finds that 48 per cent of the inmates of the reformatory have intellects ranging from three to eight years below that of the average normal adult. According to Dr. Orton, the average inmate of reformatory schools and penitentiaries today is not the vicious, desperate type, as was at one time supposed, but is rather a weakling, lacking in will power as the result of heredity or improper education. Dr. H. H. Goddard of the Training Schools for feeble-minded at Vineland, N. J., has investigated the children in the training schools, and has found, almost without exception, a history of feeble-mindedness for several generations, showing that practically all the inmates of the School for Feeble-minded are the offspring of parents who were themselves weak minded. Similar investigations carried on by Dr. David F. Weeks, superintendent of the New Jersey State Village of Epileptics, at Skillman, N. J., shows an invariable history of feeble-mindedness or degeneracy, extreme poverty on account of lack of mental and physical ability to make a living, tuberculosis, epilepsy and alcoholism in many cases, and over 30 per cent of criminals in the immediate ancestry of these patients. Regarding the physical condition of the inmates of the reformatory, Dr. Orton found that many of them were physically unable to earn an honest living. These facts he advances in support of the law recently adopted by the New Jersey Legislature, authorizing the sterilization of defective persons or confirmed criminals, so as to prevent further offspring.

---
